# Peri-Infarct Pericarditis in a Late-Presenting Myocardial Infarction

**DOI:** 10.7759/cureus.71163

**Published:** 2024-10-09

**Authors:** Michael N Zarrella, Phelese Smith, Basel Saadeh, Ashish Arora

**Affiliations:** 1 Internal Medicine, Saint Mary's Hospital, Waterbury, USA; 2 Critical Care Medicine, Saint Mary's Hospital, Waterbury, USA

**Keywords:** chest pain, colchicine, pericarditis, peri-infarct pericarditis, st-elevation myocardial infarction (stemi)

## Abstract

Recognizing a myocardial infarction (MI) is an important requirement of any clinician; however, what may become challenging is recognizing post-MI-related complications. In cases such as the one presented here, some patients who are late to receive evaluation with MI can develop peri-infarct pericarditis. This unique complication is important to consider should patients develop signs of pericarditis after a recent MI and requires a treatment regimen designed to manage both pericarditis and MI simultaneously.

## Introduction

Peri-infarct pericarditis (PIP) is a complication that commonly occurs within three days following a myocardial infarction (MI) and is thought to be caused by an inflammatory reaction to transmural ischemia [1,2]. PIP presents with at least two of four features typical of acute pericarditis such as characteristic chest pain that is relieved leaning forward, pericardial friction rub on exam, pericardial effusion, and associated changes on electrocardiogram (EKG) typically diffuse PR-segment depression and ST-segment elevation [3]. While not often affecting mortality or morbidity, it is important to note that the clinical syndrome itself can be debilitating for patients [3]. Incidence rates of PIP are fairly low, with limited evidence-based literature available other than a small number of case reports such as the one presented here. One study out of Israel which evaluated ST-segment elevation MI patients over a 13-year period demonstrated only 1.2% of patients developed PIP [1]. Rates of occurrence of PIP have decreased drastically in the era of percutaneous coronary intervention [4]. However, despite its low incidence rates in recent years, it is important to consider PIP in patients with recent MI who develop signs of PIP so that it may be appropriately managed. 

## Case presentation

A 47-year-old man presented to the emergency department with complaints of constant, severe left-sided chest pain radiating from his chest wall to his left shoulder and arm that began yesterday while moving furniture. His past medical history was significant for hypertension, hyperlipidemia, and cigarette smoking, and he had a brother who had an MI at the age of 38. 

**Figure 1:**
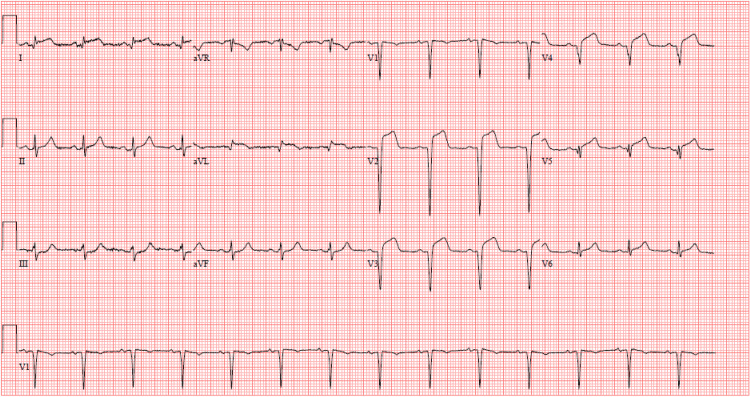
EKG: anterolateral STEMI ST-segment elevations in leads I, aVL, and V2-V6, with reciprocal ST-segment depressions in inferior leads III and aVF representing an anterolateral myocardial infarction EKG: electrocardiogram; STEMI: ST-segment elevation myocardial infarction

The physical exam was negative for chest tenderness, murmurs, rubs, or gallops, and the respiratory exam was clear to auscultation. Ancillary data was remarkable for high-sensitivity troponin I elevation greater than 22,973 nanograms per liter (ng/L) (reference range: 0-39 ng/L) and subsequent EKG revealing ST-segment elevations in anterior and lateral leads. The patient was diagnosed with an ST-segment elevation myocardial infarction (STEMI) (Figure 1). 

The patient was immediately sent for cardiac catheterization which revealed complete occlusion of the proximal left anterior descending artery managed with the placement of two drug-eluting stents followed by the initiation of dual antiplatelet therapy.

**Figure 2:**
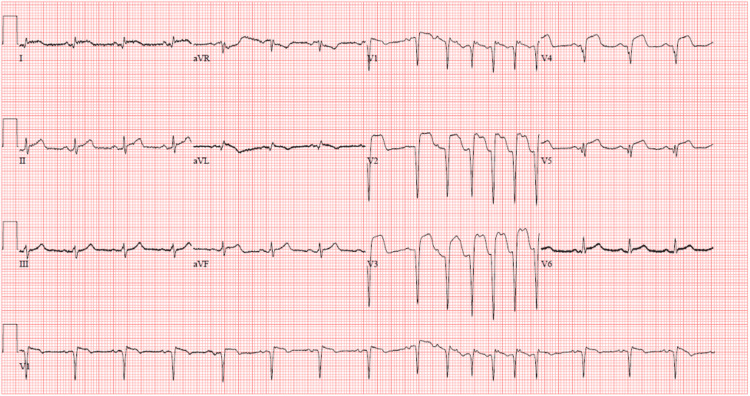
Repeat EKG Forty-eight hours post-percutaneous intervention, ST-segment elevations remain persistent in anterolateral leads, but are newly developing in inferior leads II and aVF STEMI: ST-segment elevation myocardial infarction

The following day, the patient complained of persistent chest pain that was left-sided and exacerbated by deep inspiration and lying flat while being relieved when leaning forward. The echocardiogram did not reveal pericardial effusion. Repeat EKG demonstrated diffuse ST-segment elevations throughout anterior, lateral, and inferior leads as shown in Figure 2.

Due to characteristic chest pain and EKG changes, the patient was diagnosed with PIP in the setting of a late-presenting STEMI. Treatment was initiated with high-dose aspirin 650 mg three times per day for a total of seven days to be followed by 81 mg daily indefinitely. Additionally, the patient was started on colchicine 0.6 mg daily for a scheduled three-month period of time. At one-month follow-up, the patient's chest pain had resolved.

## Discussion

The likelihood of post-infarct pericarditis is influenced by various factors, with the location of the infarction being a significant determinant, as a higher incidence of cases was observed after anterior infarctions [1,2]. Patients who develop PIP often exhibit an ejection fraction below 50% and markedly elevated troponin levels [1,2]. Although not specifically present in this case, pericardial effusions frequently accompany PIP cases, contributing to increased rates of both mortality and morbidity [4]. Therefore, clinicians should consider PIP in patients with persistent chest pain post-MI for prompt diagnosis and management [3,4]. Diagnosing PIP is similar to that of acute pericarditis as mentioned earlier; however, it varies in that it occurs directly following an MI.

PIP is usually self-limited, but recommendations are to avoid non-steroidal anti-inflammatory drugs (NSAIDs) [4]. Similarly, it is recommended to avoid glucocorticoids as they may prolong the disease course and are associated with high rates of recurrence [3,4]. For patients with significant symptoms who require analgesia, acetaminophen should be used as an initial treatment. If the patient remains symptomatic, high-dose aspirin defined as 650 mg every six to eight hours is recommended rather than other NSAIDs [4]. It should be noted, however, that there is no current evidence to suggest using aspirin or other NSAIDs will improve outcomes [4].

Unlike post-cardiac injury syndromes, there is a lack of published data on the utilization of colchicine in treating PIP, mainly because very few cases progress in severity with the continued use of aspirin post-acute MI and the diagnosis is often missed [4]. There remains data to suggest that the addition of colchicine may actually be beneficial for these patients.

In evaluating the use of colchicine post-acute MI, the COLCOT trial reveals a 23% relative risk reduction in cardiovascular events with initiating colchicine within 30 days post-MI when compared to placebo [5]. While the CONVERT-MI trial did not reveal a benefit post-MI, it is important to note that the subgroup analysis in the COLCOT trial initiated colchicine within three days post-acute MI [5,6]. This warrants not only further investigation into the use of colchicine post-MI in reducing cardiovascular events but also more research in assessing the prevention of PIP post-acute MI and to provide more solid data for the opportunity to devise guidelines outlining optimal dose, duration, and timing of initiating colchicine post-acute MI [7].

## Conclusions

Encountering an MI is fairly common in inpatient practice, and due to modern cardiovascular practices, it is less common to see patients who develop PIP. However, it remains important to be able to recognize and manage PIP due to the symptomatic burden it can have on patients. There remains mixed evidence-based practice with regard to a fixed treatment regimen for PIP, but as demonstrated in this case, a course of high-dose aspirin in addition to colchicine was an effective clinical management. Future considerations for randomized controlled trials or meta-analyses may be helpful in creating standardized practice for patients who develop PIP.
